# The concentration of resistin in perivascular adipose tissue after CABG and postoperative atrial fibrillation

**DOI:** 10.1186/s12872-019-1254-5

**Published:** 2019-12-16

**Authors:** Maciej Rachwalik, Marta Obremska, Dorota Zyśko, Małgorzata Matusiewicz, Krzysztof Ściborski, Marek Jasiński

**Affiliations:** 1 Department and Clinic of Cardiac Surgery, Wroclaw Medical University, ul. Borowska 213, 50-556 Wrocław, Poland; 2 Department of Emergency Medical Service, Wroclaw Medical University, Wroclaw, Poland; 3 Department and Clinic of Emergency Medicine, Wroclaw Medical University, Wroclaw, Poland; 4 Department of Medical Biochemistry, Wroclaw Medical University, Wroclaw, Poland; 5 Department and Clinic of Cardiology, Wroclaw Medical University, Wroclaw, Poland

**Keywords:** Resistin, Postoperative atrial fibrillation, Coronary artery bypass graft

## Abstract

**Background:**

Postoperative atrial fibrillation occurs in up to 30% of patients after coronary artery bypass graft (CABG) and its cause is unknown. The aim of the study was to evaluate whether concentration of resistin in surrounding coronary artery perivascular adipose tissue (PVAT) is related to postoperative atrial fibrillation occurrence.

**Methods:**

A total number of 46 patients (35 male, 11 female; median age 66.5) were qualified for elective CABG. Medical history, laboratory test results and echocardiographic parameters were noted. Patients were monitored up to 3 days after CABG and then were divided into groups with and without postoperative atrial fibrillation occurrence. Fragments of PVAT were collected intra-operatively: near the left anterior descending artery and main left coronary artery. The concentration of resistin was determined by Human Resistin Quantikine ELISA Kit and expressed as ng/g. A multivariate stepwise logistic regression analysis was performed to find variables related to postoperative atrial fibrillation occurrence.

**Results:**

Postoperative atrial fibrillation occurred in 14 (30.4%) patients. The patients with and without postoperative atrial fibrillation were similar in age, gender, epicardial adipose tissue thickness and laboratory parameters. The concentration of resistin in PVAT near the left main coronary artery was significantly higher in patients with postoperative atrial fibrillation than in those without the complication (*P* = 0.03). In the multivariate stepwise logistic regression analysis the concentration of resistin above cut-off point 54 ng/g in PVAT near left main coronary artery was independently related to postoperative atrial fibrillation occurrence (OR: 7.7; 95% CI:1.4–42.2 *p* = 0.02).

**Conclusions:**

The higher concentrations of resistin in PVAT near the left main coronary artery which is located close to the left atrium are associated with postoperative atrial fibrillation.

## Background

Atrial fibrillation is a common cardiac arrhythmia and is associated with elevated morbidity and mortality [1]. This also applies to atrial fibrillation after cardiac surgery as postoperative atrial fibrillation [2]. It is estimated that de novo postoperative atrial fibrillation may occur in up to 30% of patients undergoing cardiac surgery. This complication often prolongs hospitalization [3]. Moreover, it can increase the risk of stroke, bleeding, infections and renal failure [4]. Postoperative atrial fibrillation may also influence post-operative long term results. It can lead to a 2-fold increase in all-cause 30-days, 6-months mortality after surgery compared to patients with sinus rhythm [5]. The rate of postoperative atrial fibrillation after cardiac surgery is much higher than the rate of atrial fibrillation after non-cardiac surgery. The causes of postoperative atrial fibrillation are multifactorial and not fully understood. Some of them depend on comorbidities of patients, others are related to the operative trauma. Recent studies suggest pro-inflammatory factors to be related to the development of postoperative atrial fibrillation [6]. An increasing number of scientific reports shows that epicardial adipose tissue is a source of inflammatory factors [7, 8]. Epicardial adipose tissue is located between the myocardium and the visceral layer of the pericardium. Perivascular adipose tissue (PVAT) is a part of epicardial adipose tissue close to coronary arteries which surrounds the coronary artery tree, while pericardial fat is a part of intrathoracic visceral adipose tissue is located to the outside of the parietal layer of the pericardium. The physiological functions of epicardial adipose tissue are storage of lipids as an energy supply for cardiomyocytes, protection of autonomic ganglia and nerve tissue and regulation in adjusting the diameter and flow in coronary vessels. Moreover, epicardial adipose tissue releases paracrine modulators of inflammatory and oxidative stress [8]. Therefore it cannot be ruled out that local factors are involved in the occurrence of postoperative atrial fibrillation. Such factors could be adipocytokines secreted by PVAT by unknown mechanisms possibly including non-specific activation of inflammation associated with perioperational stress. It is not known whether increased resistin concentration in PVAT is related to postoperative atrial fibrillation.

The aim of the study is to assess whether of PVAT resistin concentration in patients undergoing coronary artery bypass grafting (CABG) is associated with the occurrence of postoperative atrial fibrillation.

## Methods

### Study design

The patients qualified for elective CABG were invited to participate in the study. The inclusion criteria were willingness to participate in the study, age < 80 years, sinus rhythm prior to the surgery, ejection fraction of the left ventricle >30%, the presence of advanced coronary artery disease (with involvement of the main trunk of left artery or two to three large arteries), and the absence of moderate or severe valvular pathology.

Exclusion criteria were thyroid diseases, ejection fraction of left ventricle less than 30%, moderate or severe valvular diseases and persistent atrial fibrillation.

The study was approved by the local bioethics committee and received the number- KB 392/2016.

All patients signed an informed consent to participate in the study. Ultimately the study group consisted of 46 patients (35 male, 11 female; aged 66.5).

**Fig. 1:**
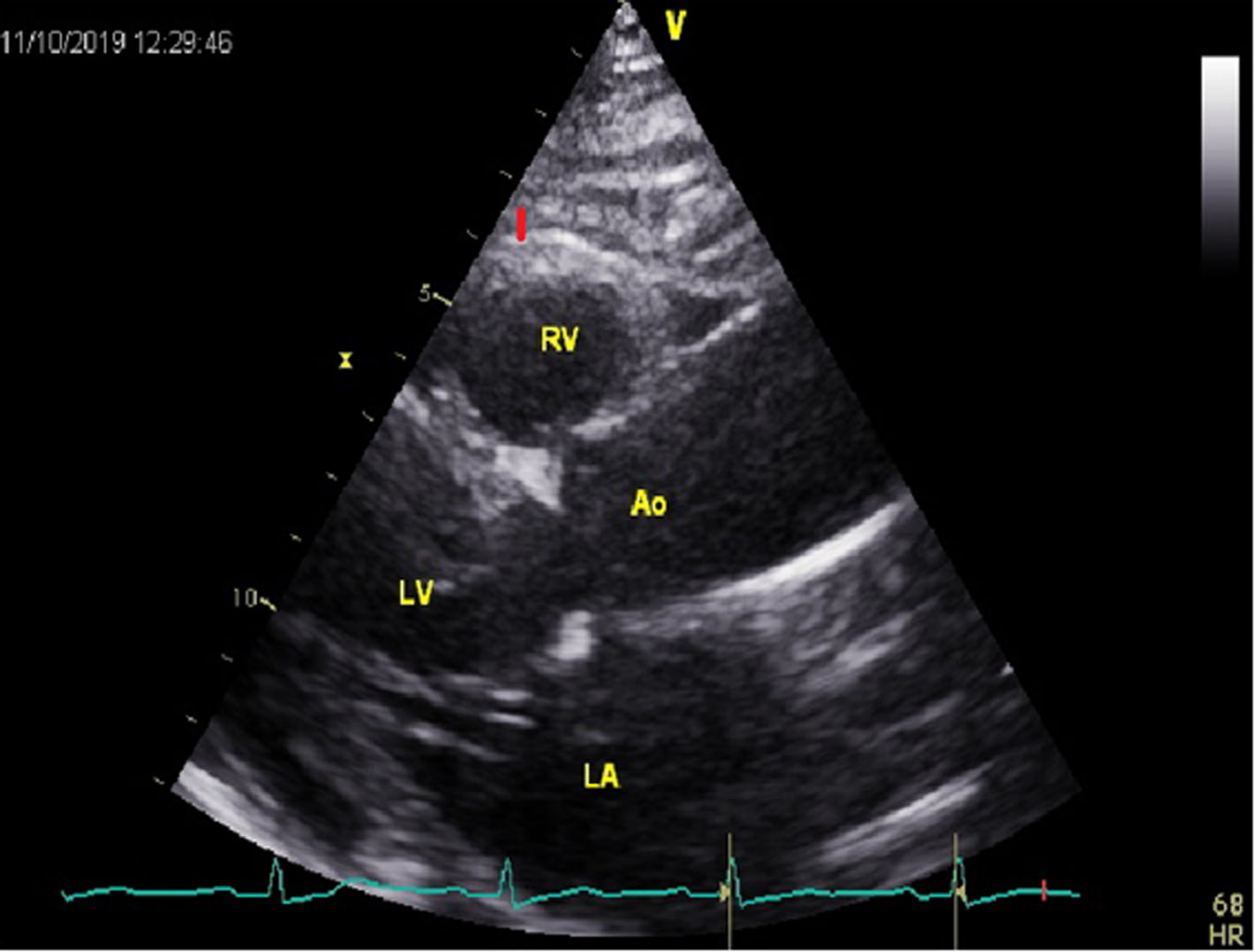
The measurement of thickness. The measurement of thickness of epicardial adipose tissue in parasternal long axis view (red segment). LV-left ventricle, Ao-aorta, LA-left atrium, RV-right ventricle.

The demographics, medical history regarding concomitant diseases: previous myocardial infarction, diabetes, hypertension, chronic obstructive pulmonary disease, renal insufficiency, and history of paroxysmal atrial fibrillation were obtained from all patients. Before surgery all patients had assessed levels of glycated haemoglobin, glucose, insulin, glomerular filtration rate (GFR), and electrolytes. The concomitant medication with beta-blockers, digoxin, angiotensin-converting enzyme inhibitor, amiodarone was noted. For each patient CHADS2-VASc score was calculated. Echocardiography was performed by the same cardiologist using GE VIVID-E 9 (Horten, Norway) cardiac ultrasound system with 2.5-to 3.5 – MHz transducer. In echocardiography we evaluated end-diastolic and end-systolic diameter of left ventricle, ejection fraction of left ventricle, end-diastolic diameter of posterior wall and intraventricular septum and volume of left atrium which was indexed by body surface area (BSA). The epicardial adipose tissue thickness was measured in each patient from the parasternal long axis view. The measurements were performed at a point on the free wall of the right ventricle at end-systole, in axis perpendicular to the aortic valve, were the thickness of epicardial adipose tissue is the highest (Fig. 1). The presence of severe right coronary artery stenosis (at least 90%) was noted.

In each patient a 12-lead electrocardiogram (ECG) was performed before initiation of surgery. ECG of all patients was monitored in the postoperative period at the intensive care unit as well as at the cardiac surgery department during 3 days after CABG. During the next 4 days resting ECG was performed daily and in the event of abnormal rhythms or malaise. Atrial fibrillation was defined according to the Guidelines for the management of atrial fibrillation of European Society of Cardiology published in 2016. The patients were divided into the group with postoperative atrial fibrillation – POAF(+) group, and to the group without postoperative atrial fibrillation – POAF(−) group.

Surgical approach and sampling.

Surgery was performed in a typical fashion, with the use of cardiopulmonary bypass. Cardioprotection was achieved by antegrade, cold blood cardioplegia. Each patient had pedicled internal thoracic artery and saphenous vein harvested. During surgery, on average 2.6 grafts were performed (range 2–3). The time of cardiopulmonary bypass and the time of cross-clamp were noted.

Fragments of PVAT were collected intra-operatively from the surface of the left ventricle after stabilization of the patient on extracorporeal circulation. Fragments of tissues (3mmx3mmx3mm) were harvested using a surgical blade without using electrocoagulation, to minimize the risk of vessel injury. There were two harvest points: in the area of the left main trunk near the appendage of the left atrium and at the distal part of the left anterior descending coronary artery near to the apex of left ventricle. Samples were immediately frozen at − 80°C and stored for further processing.

### Laboratory methods

Tissue samples were homogenized in a buffer containing PMSF (phenylmethylsulfonyl fluoride) using FastPrep homogenizer (MP Biomedicals, Santa Ana, CA, USA) and centrifuged for 10 min at 14,000 g at 4°C. Supernatants were stored at − 80°C until analyse. Just before the analysis, the supernatants were centrifuged again for 10 min at 4°C at 14,000 g. The concentration of resistin was determined using commercially available Human Resistin Quantikine ELISA Kit from R&D Systems (Minneapolis, MN, USA) in accordance with manufacturer procedure and was expressed as ng/g of tissue. For ELISA kit Intra-Assay coeficcient of variance (CI) was 3.8–5.3% (20 measurements in the same sample with known concentration - 3 concentrations), depending on the concentration, and for Inter-Assay: 7.8–9.2% (40 measurements using different sets made by 3 technicians) depending on the concentration.

### Statistical analysis

The continuous variables were presented as means and standard deviations and were compared with Student T test, Mann Whitney U test according to their distribution, and discrete variables were presented as numbers and percentages and compared with chi2 test Receiver operating characteristic curve (ROC) analysis was performed to identify cut-off point of the concentration of resistin to predict postoperative atrial fibrillation occurrence.

The multivariate stepwise logistic regression analysis was performed to find variables related to postoperative atrial fibrillation occurrence. The factors which in univariate analysis differ between groups with *P* less than 0.15 or were clinically important, and concentration of resistin dichotomized according to the cut-off values assessed in ROC analysis were used as independent variables. *P* less than 0.05 were considered statistically significant.

## Results

Postoperative atrial fibrillation occurred in 14 (30.4%) patients after CABG while in 32 (69.6%) patients postoperative atrial fibrillation was not observed.

A total of 4 (8%) patients had paroxysmal atrial fibrillation in medical history before CABG and all of them proceeded to have postoperative atrial fibrillation, therefore paroxysmal atrial fibrillation in medical history could not be taken into multivariate regression analysis. Concomitant medication was presented in Table 1. No patient had received digoxin, and amiodarone was only administered after an episode of postoperative atrial fibrillation.

**Table 1:** The demographic, clinical, and laboratory parameters of patients in both groups

	POAF group (*n* = 14)	No POAF group (*n* = 32)	*P*
Gender male N (%)	11 (78.6)	24 (75.0)	0.80
Age, years X ± SD	64.2 ± 6.3	67.5 ± 9.5	0.25
BMI, kg/m2 ± SD	28.6 ± 3.7	29.1 ± 4.5	0.72
Diabetes N (%)	2 (14.3)	16.0 (50)	0.02^a^
Hypertension N (%)	12 (85.7)	27 (84.4)	0.91
Previous myocardial infraction N (%)	5 (35.7)	10 (31.3)	0.77
Previous PCI N (%)	2 (14.3)	3 (9.4)	0.62
Stroke N (%)	2 (14.3)	0 (0)	0.18
Paroxysmal AF in history N (%)	4 (28.6)	0 (0)	0.01^a^
Waist, cm ± SD	100.5 ± 10.5	99.1 ± 10.3	0.68
GFR < 40, ml/min N (%)	3 (21.4)	0 (0)	0.05^a^
HbA1c, mmol/l ± SD	6.1 ± 0.6	6.3 ± 1.0	0.51
Glucose, mg% ± SD	113.2 ± 47.2	119.9 ± 45.8	0.66
Insulin, uU/mL ± SD	16.1 ± 8.6	24.2 ± 25.4	0.25
CHA2DS2VASc score ± SD	2.6 ± 1.4	2.9 ± 1.1	0.51
Betablockers N (%)	12 (85.7)	26 (68.4)	0.71
ACEI N (%)	9 (64.3)	15 (46.9)	0.28
Aortic cross clamp time, minutes±SD	40.5 ± 11.0	42.3 ± 10.6.	0.61
Cardiopulmonary bypass time, minutes±SD	78.4 ± 19.2	79.0 ± 17.2	0.92
COPD N (%)	0 (0)	1 (3.1)	0.67
Stenosis of RCA ≥ 90% N(%)	8 (57.1)	12 (37.5)	0.20
Potassium level, mEq/l ± SD	4.1 ± 0.4	4.2 ± 0.4	0.43
Magnesium level, mEq/l ± SD	2.1 ± 03	2,1 ± 0,3	0.93

*ACEI* angiotensin-converting-enzyme inhibitor; *AF* atrial fibrillation; *BMI* body mass index; *CHA2DS2VASc* score for atrial fibrillation stroke risk calculator; *COPD* chronic obstructive pulmonary disease; *GFR* glomerular filtration rate; *HbA1c* glycated haemoglobin; *POAF* Postoperative atrial fibrillation; *N* number of patients, *RCA* right coronary artery; *X* mean value, *SD* standard deviation

^a^Statistically significant

There were no differences between the POAF(+) group and the PAOF(−) group in terms of age, gender, body mass index (BMI) and waist circumference. Similarly, both groups did not differ in laboratory measurements. The history of hypertension occurrence, chronic obstructive pulmonary diseases, previous myocardial infarction, previous percutaneous coronary intervention, and stroke were similar in both groups. Diabetes mellitus was more frequent in POAF(−) group (*P* = 0.02). The CHA2DS2VASc score was similar in both groups. All demographic, clinical, and biochemical parameters are presented in Table 1.

The echocardiographic measurements of left ventricle and left atrium were similar in both groups. The thickness of epicardial adipose tissue was similar in both groups. All echocardiographic parameters are shown in Table 2.

**Table 2:** The echocardiographic parameters in both groups

	POAF(+) (*n* = 14) X ± SD	POAF(−) (*n* = 32) X ± SD	*P*
LVEDd, mm	48.1 ± 5.2	49.9 ± 4.5	0.5
LVESd, mm	31.9 ± 4.7	32.6 ± 4.8	0.99
IVSEDd, mm	12.5 ± 1.7	11.9 ± 2.4	0.2
PVEDd, mm	11.3 ± 1.1	10.7 ± 1.1	0.98
LAVI	37.4 ± 6.5	37.3 ± 5.4	0.97
EF 30–45% N	3 (21.4)	3 (9.4)	0.26
EF	52.9 ± 10.1	56.1 ± 6.7	0.07
EAT, mm	7.1 ± 1.2	7.4 ± 1.2	0.39

*EAT* epicardial adipose tissue, *EF* ejection fraction of left ventricle, *IVEDd* end-diastolic diameter of the intraventricular septum, *LAVI* indexed volume of left atrium, *LVEDd* end-diastolic diameter of the left ventricle, *LVESd* end-systolic diameter of the left ventricle, *mm* millimetres, *N/n* number of patients, *POAF(+)* patients with postoperative atrial fibrillation; *POAF(−)* patients without postoperative atrial fibrillation, *PWEDd* end-diastolic diameter of the posterior wall, *X* mean value, *SD* standard deviation

*statistically significant

The resistin concentration in PVAT at the distal part of the left anterior descending coronary artery was higher in the postoperative atrial fibrillation group but it was not found to be statistically significant (*P* = 0.15). In PVAT close to the left main trunk resistin concentration was significantly higher in the POAF(+) than in the POAF(−) group (*P* = 0.03). There were no statistical differences in resistin concentrations between PVAT near to the trunk of left coronary artery and PVAT near to the distal part of left descending artery in each of the studied groups (Table 3).

**Table 3:** The concentration of resistin in perivascular adipose tissue in two regions in the heart surface in both groups

	POAF(+) (*n* = 14) X ± SD	POAF(−) (*n* = 32) X ± SD	P
PVAT-LAD, ng/g	145.7 ± 184.6	96.6 ± 154.7	0.15
PVAT-LM, ng/g	166.6 ± 165.1	83.6 ± 101.0	0.03^a^
P	0.47	0.99	

*PVAT –LAD* perivascular adipose tissue in the area of the left anterior descending artery, *PVAT-LM* perivascular adipose tissue in the area of the left main trunk, *n* number of patients, *POAF(+)* patients with postoperative atrial fibrillation; *POAF(−)* patients without postoperative atrial fibrillation, *X* mean value, *SD* standard deviation

^a^Statistically significant

**Fig. 2:**
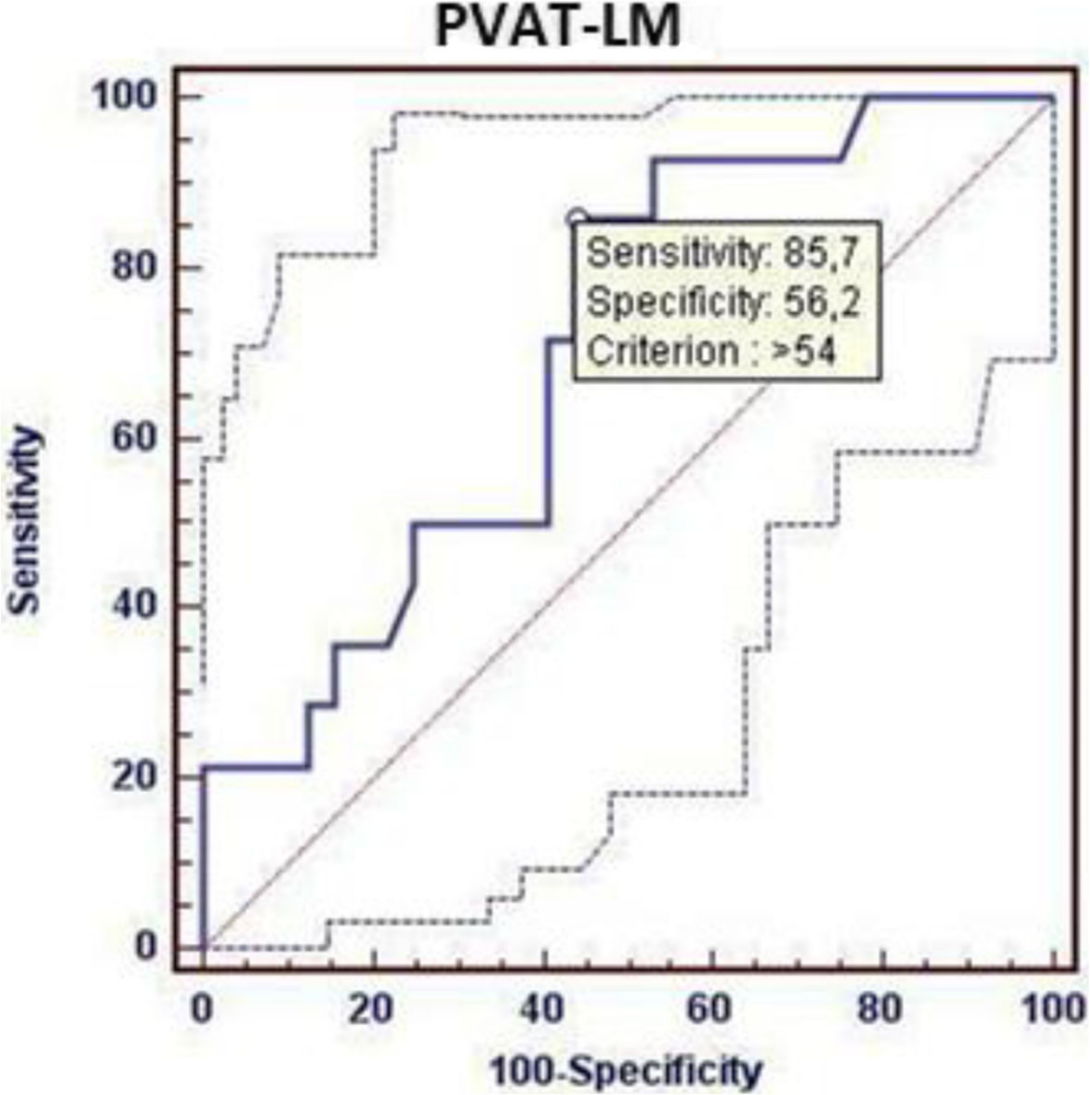
ROC curve. The cut off point (54 ng/g) for the concentration of resistin which has the best sensitivity and specificity. to predict POAF occurrence. AUC 0.703, CI:0.550–0.829 p0.022. PVAT-LM - perivascular adipose tissue in the area of the left main trunk.

The cut off point for the concentration of resistin which has the best sensitivity and specificity to predict postoperative atrial fibrillation occurrence was determined at 54 ng/g. (Fig. 2).

The multivariate stepwise logistic regression analysis revealed that only resistin concentration in PVAT close to the left main trunk higher than 54 ng/g is related with postoperative atrial fibrillation occurrence (OR: 7.7; 95% CI 1.4–42.2 *p* = 0.02).

Because all patients who had atrial fibrillation before surgery had postoperative atrial fibrillation this variable could not be used in multivariate stepwise logistic regression analysis.

## Discussion

The main finding of the study is that higher concentration of resistin close to the left main trunk is associated with the occurrence of postoperative atrial fibrillation.

Resistin is known to be a proinflammatory cytokine and many studies indicate a relationship between higher plasma concentration of resistin and occurrence of atrial fibrillation [9]. The Framingham Offspring Study showed that higher plasma levels of resistin were related to higher incidence of atrial fibrillation in the general population [10]. This relation remained present after adjusting for traditional atrial fibrillation risk factors. Activation of inflammation may account for this relation [11, 12]. Increased resistin level is associated with higher levels of other inflammatory markers such as C-reactive protein, interleukins, tumor necrosis factor [8, 13–15].

The role of inflammation in the development of postoperative atrial fibrillation was shown by Gungor et al. [16]. They examined 40 patients after CABG and revealed that postoperative elevated C-reactive protein and resistin serum levels were significantly higher in patients with postoperative atrial fibrillation. Moreover, in this study, only serum resistin level was the independent factor of atrial fibrillation after CABG. Laurikka et al. analysed serum resistin levels in patients undergoing CABG and indicated correlation with an oxidative stress and myocardial injury [17]. The level of plasma resistin increased after CABG and maximum value was observed 24 h after surgery and correlated with increased levels of myeloperoxidase and troponins.

The epicardial adipose tissue including its perivascular part is a source of inflammatory cytokines which can diffuse into the adjacent myocardium and accumulate in the pericardial fluid. In previous studies pericardial fat and epicardial adipose tissue were not distinguished and an association between them and occurrence of atrial fibrillation was demonstrated [18, 19]. In the Framingham Heart study in the general population epicardial adipose tissue volume quantified by computed tomography (CT) was an independent predictor of atrial fibrillation [20]. The authors indicate the importance of the contiguity of the epicardial adipose tissue to atrial tissue in the genesis of atrial fibrillation. The positron emission tomography (PET) imaging is considered as a gold standard in noninvasive imaging of tissue inflammation. Using PET Mazurek et al. showed the greater inflammatory activity of epicardial adipose tissue adjacent to the left atrium, atrioventricular groove, and also left the main artery as compared to the subcutaneous or visceral thoracic tissue in patient with history of atrial fibrillation [21]. Similar findings observed in patients undergoing CABG. Drossos et al. reported that pericardial fat volume measured by CT is a strong predictor of postoperative atrial fibrillation in patients after elective surgical revascularization [22]. Similarly Opolski et al. found that an extent of the epicardial adipose tissue volume surrounding the left atrium from coronary CT angiography data sets independently predicted the incidence of postoperative atrial fibrillation [23]. These studies correspond to the result of our study. It could be noted that the PVAT near the distal part of the left anterior descending coronary artery is located close to the muscle of the ventricles while PVAT to the left main trunk is located close to the atria. The proximity of PVAT with atrial tissue seems to be crucial to the spread of inflammation. The concept of peri-atrial adipose tissue is evolving. Nakanishi et al. measured peri-atrial epicardial adipose tissue volume by multidetector CT in patient with coronary diseases and demonstrated its association with the occurrence of nonvalvular atrial fibrillation independent of the presence of hypertension, diabetes or the left atrium enlargement [24]. Such association was not observed for total epicardial adipose tissue volume. This study could indicate” on outside to inside” effect of adipose tissue on electrical and structural remodelling of left atrium. The atypical character of peri-atrial epicardial adipose tissue was also confirmed by Gaborit et al. [25]. They showed unique transcriptomic characteristics of peri-atrial epicardial adipose tissue with expression of genes implicated in oxidative phosphorylation, muscular contraction, and calcium signalling.

Numaguchi et al. in a study regarding atherosclerosis showed pronounced chronic inflammation and adipose tissue remodeling in PVAT surrounding left coronary artery compared with PVAT surrounding internal thoracic artery and subcutaneous fat pad of patients who underwent elective CABG surgery [26]. The study indicated differential phenotypes and functions of PVAT in various areas of body.

Another result of the study is that the epicardial adipose tissue assessed by echocardiography is not significantly different between the groups. Echocardiography measurements of epicardial adipose tissue are not volumetric because they assess only its thickness. Moreover it is impossible to assess PVAT by this modality due to overlapping with epicardial adipose tissue. Echocardiographic measurement of epicardial adipose tissue remains an important method for cardiovascular risk stratification in the general population. Many studies demonstrated a relationship between epicardial adipose tissue, coronary atherosclerosis, and cardiac disease such as atrial fibrillation. However quantification of epicardial adipose tissue is not included in the recommended algorithms for this risk stratification. In our study all patients had advanced coronary atherosclerosis and probably due to this disease enlarged thickness of epicardial adipose tissue, Artificial intelligence-based image analysis probably provide accurate measurement of PVAT [27].

The obtained results indicate the regional differences in the concentration of resistin in PVAT. The stress of surgery does not lead to the onset of postoperative atrial fibrillation in all patients after CABG. This observation suggests that the concentration of resistance in PVAT appears to be more important than the total amount of epicardial adipose tissue for postoperative atrial fibrillation.. Higher concentration of resistin near the trunk of left coronary artery is related with occurence of postoperative atrial fibrillation. It highlights the importance of PVAT proximity to the left atrium and thus the possibility of its electrical remodeling by proinflammatory factors. The importance of proximity of PVAT to the left atrium for occurrence of atrial fibrillation was highlighted in non-invasive studies [20–23].

Resistin levels increased in both PVAT and serum due to still unknown factors. Plasma resistin levels are derived from many areas of adipose tissue due to many reasons including surgical stress, but it does not lead to postoperative atrial fibrillation in all patients with visceral obesity. It should be noted that increased resistin level is also associated with coronary heart diseases in general population [28, 29].

During our study patients with postoperative atrial fibrillation were statistically less likely to be diabetic. This is an unexpected result because diabetes is considered to be a risk factor for atrial fibrillation occurrence. In this case it can be explained by an increased tendency to the occurrence of persistent atrial fibrillation in the group of diabetic patients and therefore a paradoxical reduction in the proportion of patients with postoperative atrial fibrillation.

Other authors found significantly higher CHA2DS2VASc and CHADS2 scores among patients with postoperative atrial fibrillation [30, 31]. In our study no,differences of the CHA2DS2VASc score between groups were observed. The differences could be related to exclusions from the study patients with low left ventricle ejection fraction which could decrease the CHADS2VASc score.

Postoperative atrial fibrillation is the result of the interaction of numerous factors that may trigger both, the initiation and continuation of this arrhythmia. The factors responsible for triggering the flickering mechanism are the subject of many works and studies. Finding the corresponding factors that may contribute to the formation of a proarrhythmogenic environment in the myocardium may create a wide field for research and potentially also have clinical implications. Our study primarily has a cognitive value indicating the local effect of resistin concentration, as a proinflammatory factor, in triggering post-operative atrial fibrillation. Epicardial adipose tissue, particularly its perivascular part located in the contiguity to the left atrium may be an environment focusing influence of many factors triggering atrial fibrillation. The increased resistin concentration in PVAT may be a mechanism by which the classical proarrhythmic factors act.

### Study limitations

This study contains several limitations. First- the study groups are relatively small and in order to further validate our report, a larger group of patients should be examined. Secondly, serum resistin was not measured. However, we focused on the differences between two regions of PVAT. Thirdly, we performed echocardiography due to its availability. The absence of significant differences between PVAT thickness in studied groups does not exclude relationships that could be demonstrated by MRI. However, most promising imaging modality is artificial intelligence MRI technics which are not introduced into clinical practice yet. Finally, patients were monitored up to the third postoperative day, at the intensive care unit and the cardiac surgery department. In future, the observation period should be extended to the entire period of hospitalization.

## Conclusions

1. Higher resistin concentration in PVAT near to the trunk of left coronary artery which is located close to the left atrium is related to atrial fibrillation occurrence after CABG.

2. The concentration of resistin in PVAT near to the distal part of left descending artery which is located close to the apex of left ventricle is not associated with atrial fibrillation after CABG.

## Data Availability

Maciej Rachwalik, Marta Obremska, Dorota Zyśko, Małgorzata Matusiewicz Krzysztof Sciborski, Marek Jasiński The datasets used and/or analysed during the current study are available from the corresponding author on reasonable request.

## Abbreviations

AF: Atrial fibrillation
BMI: Body mass index
BSA: Body surface area
CABG: Coronary artery bypass grafting
CHA2DS2VASc: Score for atrial fibrillation stroke risk calculator
COPD: Chronic obstructive pulmonary disease
GFR: Glomerular filtration rate
HbA1c: Glycated haemoglobin
IVEDd: End-diastolic diameter of the intraventricular septum
LAVI: Indexed volume of left atrium
LVEDd: End-diastolic diameter of the left ventricle
LVESd: End-systolic diameter of the left ventricle
mm: Millimetres
PVAT: Perivascular adipose tissue
EF: Ejection fraction of left ventricle
PWEDd: End-diastolic diameter of the posterior wall
POAF: Postoperative atrial fibrillation
